# Infrared Absorption Spectra of Some 1-Acetamido Pyranoid Derivatives and Reducing, Acetylated Pyranoses

**DOI:** 10.6028/jres.065A.002

**Published:** 1961-02-01

**Authors:** R. Stuart Tipson, Horace S. Isbell

## Abstract

The infrared absorption spectra of five *N*-glycopyranosylacetamides and of six acetate esters thereof are presented, together with the spectra of five related compounds, for the range of 5000 to 250 cm^−1^. Analysis of the spectra permitted assignment of characteristic group-frequencies.

For comparison, the spectra of eight reducing, pyranose acetates are also given. The general effect (on the spectra) of changing the anomeric group from hydroxyl to (a) acetamido and (b) methoxyl, when all (other) hydroxyl groups are acetylated, is pointed out.

## 1. Scope and Purpose of the Project

The main object of the present project was to record the infrared absorption spectra of the *N*-acetyl derivatives of a variety of glycopyranosylamines and their acetate esters, so that assignments of group frequencies could be made.

The spectra of five *N*-glycopyranosylacetamides and of six acetate esters thereof have been recorded, and the spectra of five related compounds have been examined. For comparison, the spectra of eight reducing, pyranose acetates are also presented.

## 2. Compounds Investigated

Table 1 gives a list of the compounds, their code numbers [1],^1^ and an index to the spectrograms; the serial number of a compound is the same as the number of its spectrogram.

The spectra were measured in the region of 5000 to 667 cm^−1^ (sodium chloride optics) and in the region of 667 to 250 cm^−1^ (cesium bromide optics). The spectrograms are given together with a discussion of (a) the structure of the compounds and (b) some of the outstanding features of their spectra.

The first 16 compounds listed in table 1 all have an *acetamido* group attached to carbon atom 1. Fourteen of them have the pyranoid ring, but two of these amides (compounds 15 and 16) are open-chain derivatives of an alditol and, presumably, have a modified form of the planar, zigzag conformation. Eight of these compounds have hydroxyl groups, and eight are acetate esters; two of the amides (compounds 3 and 13) are hydrates. Compounds 17 to 24 have a *hydroxyl* group attached to the anomeric carbon atom, and all of them are acetate esters of pyranoid sugars.

The seven pyranoid 1-acetamido compounds having free hydroxyl groups differ in regard to one or more of the following structural features: (a) The *α* or *β* anomeric configuration at carbon atom 1; (b) the configurations of the other carbon atoms of the pyranoid ring (including C5 in the hexopyranosyl derivatives and hexuronamides); and (c) the nature of the substituent, if any, at C5. Similar distinctions apply to their acetate esters and to the reducing, acetylated pyranoses.

## 3. Classification of the 1-Acetamido Derivatives Into Structurally and Configurationally Related Groups

The 16 compounds (1 to 16) were divided into two structural groups, according to whether they did or did not have a pyranoid ring.

The pyranoid compounds were divided into three groups; the members of each group have like configurational features.

### 3.1. *N*-Glycopyranosylacetamides of the *xylo* Configuration

The members of this group have the following general formula (I).

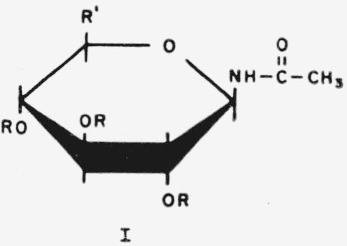



Compounds 1, 2, 6, and 7 have the above general formula, with the following substituents.

1. *N*-Acetyl-*β*-d-xylopyranosylamine, R=H; R′ = H.
2. *N*-Acetyl-*β*-d-glucopyranosylamine, R=H; R′ = CH_2_OH.
6. *N*-Acetyl-2,3,4-tri-*O*-acetyl-*β*-d-xylosylamine, R = Ac; R′=H.
7. *N*-Acetyl-2,3,4,6-tetra-*O*-acetyl-*β*-d-glucosylamine, R=Ac; R′ = CH_2_OAc.

### 3.2. *N*-Glycopyranosylacetamides of the *lyxo* Configuration

The members of this group have the following general formula (II).

Compounds 3, 12, 8, and 14 have this general formula, with the following substituents.

3. *N*-Acetyl-*β*-d-mannopyranosylamine, monohydrate, R=H; R′=CH_2_OH.
12. 1-Acetamido-1-deoxy-?-d-mannopyranuronamide, R=H; R′ = CONH_2_, and the disposition at C1 *may* be the opposite of that depicted.
8. *N*-Acetyl-2,3,4,6-tetra-*O*-acetyl-*β*-d-mannosylamine, R=Ac; R′=CE_2_OAc.
14. 1-Acetamido-2,3,4-tri-*O*-acetyl-1-deox**y-?**-d-mannuronamide, R=Ac; R′=CONE_2_, and the disposition at C1 *may* be the opposite of that depicted.

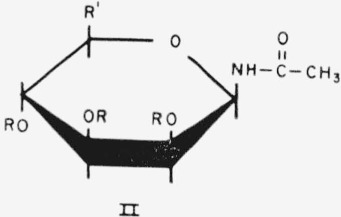

### 3.3. *N*-Glycopyranosylacetamides of the *arabino* Configuration

The members of this group have the following general formula (III).

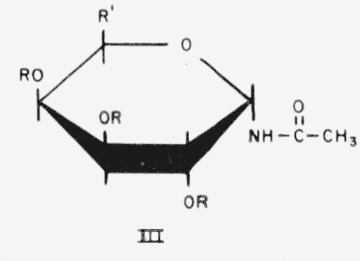



Compounds 10 and, possibly, 13 have the above general formula, with the following substituents.

10. *N*-Acetyl-2,3,4,6-tetra-*O*-acetyl-*α*-d-galactosylamine, R=Ac; R′ = CH_2_OAc.
13. 1-Acetamido-1-deox-*α*(?)-d-galactopyranuronamide pentallydrate, R=H; R′ = CONH_2_.

Compounds 4, 5, 9, and 11 (and, possibly, 13) have formula IV, with the substituents indicated.

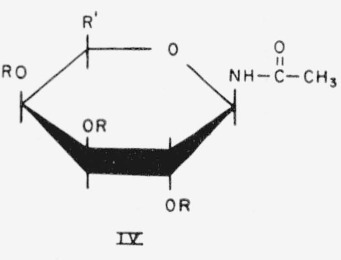

4. *N*-Acetyl-*α*-l-arabinopyranosylamine, R=H; R′=H.
5. *N*-Acetyl-*β*-d-galactopyranosylamine, R=H; R′ = CH_2_OH.
9. *N*-Acetyl-2,3,4-tri-*O*-acetyl-*α*-l-arabinosylamine, R=Ac; R′ = H.
11. *N*-Acetyl-2,3,4,6-tetra-*O*-acetyl-*β*-d-galactosylamine, R=Ac; R′ = CH_2_OAc.
13. 1-Acetamido-1-deoxy-*β*(?)-d-galactopyranuronamide pentahydrate, R=H; R′ = CONH_2_.

### 3.4. 1,1-Bis(acetamido)-1-deoxypentitol Derivatives

Compounds 15 and 16 have the general formula V, with the following substituents.

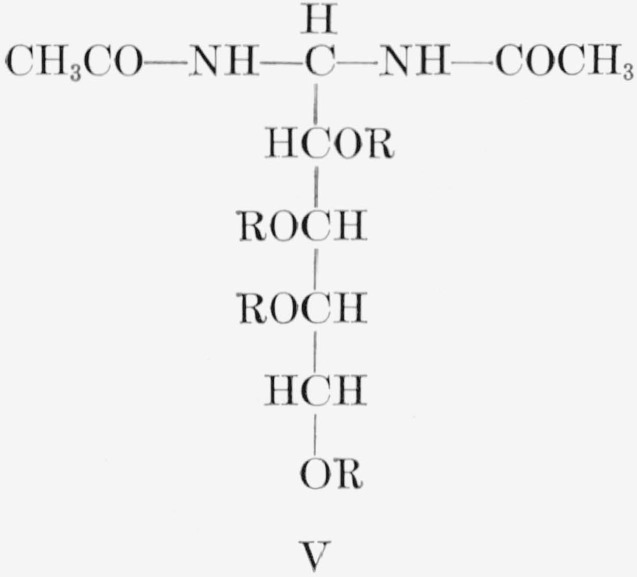

15. 1,1-Bis(acetamido)-1-deoxy-l-arabinitol, R = H.
16. 1,1-Bis(acetamido)-2,3,4,5-tetra-*O*-acetyl-1-deoxy-l-arabinitol, R=Ac.

These compounds probably adopt a modified form of the planar, zigzag conformation depicted in general formula VI.

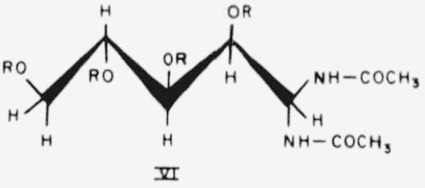



## 4. Classification of the Reducing, Acetylated Pyranoses Into Structurally and Configurationally Related Groups

These eight pyranoid acetates (compounds 17 to 24) were divided into four configurational groups. Three of the compounds (18, 20, and 22) are ketose derivatives.

### 4.1. Reducing, Acetylated Pyranoses of the *xylo* Configuration

The members of this group have the following general formula (VII).

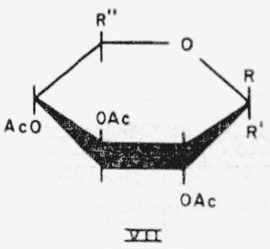



Compounds 17 to 20 have the above general formula, with the following substituents.

17. 2,3,4-Tri-O-acetyl-*α*-d-xylopyranose, R=H; R′=OH; and R″=H.
18. 1,3,4,5-Tetra-*O*-acetyl-*α*-l-*xylo*-hexulopyranose (1,3,4,5-tetra-*O*-acetyl-*α*-l-sorbopyranose), R = CH_2_OAc; R′=OH; R″ = H; and the molecule is the mirror image of that depicted.
19. 2,3,4,6-Tetra-*O*-acetyl-*β*-d-glucopyranose, R = OH; R′=H; and R″=CH_2_OAc.
20. 1,3,4,5,7-Penta-*O*-acetyl-*α*-d-*gluco*-heptulopyranose, R = CH_2_OAc; R′=OH; and R″ = CH_2_OAc.

### 4.2. Reducing, Acetylated Pyranose of the *lyxo* Configuration

Compound 21 has the following formula (VIII).

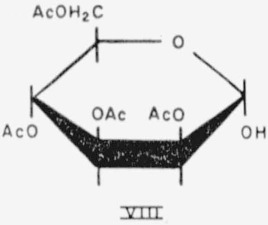

21. 2,3,4,6-Tetra-*O*-acetyl-*α*-d-mannopyranose

### 4.3. Reducing, Acetylated Pyranoses of the *arabino* Configuration

These compounds have the following general formula (IX).

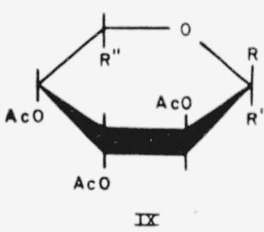

22. 1,3,4,5-Tetra-*O*-acetyl-*β*-d-*arabino*-hexulopyranose (1,3,4,5-tetra-*O*-acetyl-*β*-d-fructopyranose), R=OH; R′ = CH_2_OAc; and R″=H.
23. 2,3,4,6-Tetra-*O*-acetyl-*β*-d-galactopyranose, R = H; R′=OH; R″ = CH_2_OAc; and the molecule is the mirror image of that depicted.

### 4.4. Reducing, Acetylated Pyranose of the *ribo* Configuration

Compound 24 has the following formula (X).

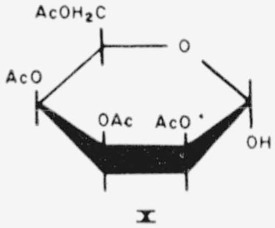

24. 2,3,4,6-Tetra-*O*-acetyl-*α*-d-talopyranose

## 5. Discussion of the Spectra

In the present study, the *positions* of the various absorption bands for each of 24 sugar derivatives have been determined; the relative intensities of absorption were not examined in detail. The bands were compiled, and were studied by statistical and comparative methods, as previously described [2].

All of the compounds in the present study *and* all 19 of the (monosaccharide) acetylated methyl aldopyranosides previously examined [3] show bands in the following spectral regions (alternative interpretations and some tentative assignments in parentheses): at 2994 to 2933 cm^−1^ (or 2976 to 2907 cm^−1^; C—H stretching); at 1449 to 1408 cm^−1^ (C—H bending); at 1379 to 1366 cm^−1^ (CH_3_—C deformation); at 1282 to 1247 cm^−1^ (acetyl, attached to nitrogen or to oxygen); at 1151 to 1114 cm^−1^; at 1107 to 1074 cm^−1^ (C—O stretching); at 1072 to 1052 cm^−1^; at 1050 to 1016 cm^−1^; at 962 to 933 cm^−1^; at 917 to 895 cm^−1^ (or 903 to 862 cm^−1^); at 636 to 600 cm^−1^ (or 625 to 597 cm^−1^; *N*-acetyl, *O*-acetyl, or both); and at 432 to 399 cm^−1^.

Except for compounds 15 and 20, all of the above compounds show a band at 1348 to 1316 cm^−1^ (C—H bending); except for compound 12, a band is shown at 1239 to 1211 cm^−1^; and, except for compounds 1, 2, and 14 to 16, a band is shown at 379 to 367 cm^−1^.

The spectra were next examined in groups, as given in table 2.

Bands characteristic of functional groups were found to fall into two categories: (a) Those that maintain their identity, and (b) those that, although present, are obscured or matched by bands (in the same spectral regions) given by compounds not possessing the functional group under consideration. Consequently, bands in the second category have no diagnostic value in the present study.

### 5.1. Bands That Maintain Their Identity

The following bands were shown *only* by compounds (in this study) having the structural features mentioned; possible assignments are given in parentheses. All of the *acetamido* compounds (group 1, table 2) showed at least one band at 3356 to 3236 cm^−1^ (N—H stretching); at 1709 to 1661 cm^−1^ (amide I); and at 1575 to 1541 cm^−1^ (amide IT). Group 1a showed a band at 3268 to 3226 cm^−1^ (or 3257 to 3215 cm^−1^); in the range of 3247 to 3205 cm^−1^, a band is also shown by nine of the other secondary amides (compounds 6 to 10, 12 to 14, and 16), but not by the members of group 5a or by any of the acetylated methyl glycosides previously studied [3]; hence, the band is presumably attributable to N—H stretching. Group 1a also showed a band at 3125 to 3077 cm^−1^, shown by three other secondary amides (compounds 6, 7, and 13), but not by any of the other compounds just mentioned; thus, this band, too, is probably attributable to N—H stretching.

All of the *acetate esters* (group 2 and the acetylated methyl glycosides) show a band at 1751 to 1736 cm^−1^ (acetate ester, C=O stretching) which is not displayed by the other compounds in this study.

### 5.2. Bands That, With These Compounds, Are Not Unequivocally Characteristic of Customarily-Assigned Features

All of the *acetamido* compounds (group 1, table 2) showed bands at 1302 to 1258 cm^−1^ (amide III) and 741 to 699 cm^−1^ (amide V), and at 1124 to 1101 cm^−1^; however, bands in one, two, or all three of these regions are also shown by some *non-nitrogenous* compounds in this study.

All of the *acetate esters* (group 2 and the acetylated methyl glycosides) show a band at 667 to 637 cm^−1^; however, a band in this region is also displayed by compounds 3 to 5 (which are not acetate esters).

For the *primary amides* (group 3), none of the bands customarily regarded as being characteristic of this functional group could be used as an unequivocal indication of its presence. These bands, shown by all the members of group 3, were as follows: At 3356 to 3322 cm^−1^ (bonded NH, N—H stretching) [shown by three of the secondary amides (compounds 9, 11, and 15)]; at 3215 to 3205 cm^−1^ (bonded NH, N—H stretching) [shown by two of the secondary amides (1 and 10)]; at 1667 to 1661 cm^−1^ (amide I) [shown by six of the secondary amides (1, 3, 4, 10, 15, 16)]; at 1445 to 1427 cm^−1^ [shown by 10 of the secondary amides (1, 2, 5 to 11, and 16), by all but one (compound 22) of the members of group 5a, and by all 24 of the methyl glycoside acetates previously studied [3]]; and at 1403 to 1391 cm^−1^ (amide VI) [shown by three of the secondary amides (4, 5, and 7), by two members of group 5a (17 and 18), and by six methyl glycoside acetates (previous study [3], compounds 2, 6, 7, 13, 22, and 23)].

The *hydrates* (group 4) show bands at 1664 to 1642 cm^−1^ that overlap, somewhat obscure, or are obscured by, amide bands in the same region.

As regards the compounds having one or more *hydroxyl groups* (group 5), all of the compounds having an anomeric hydroxyl group only (group 5a) show a band at 3597 to 3367 cm^−1^ (O—H stretching) not displayed by any acetylated methyl glycoside studied [3]. However, a band in this region is shown by the amides 2, 3, 5, 12, 13, and 15 (having hydroxyl groups) *and* by the amides 8, 11, 14, and, perhaps, 16 (compounds having no hydroxyl group); for these compounds, the band is, presumably, attributable to N—H stretching. Furthermore, as previously mentioned, all of the compounds in the present study (and 19 acetylated methyl aldopyranosides) show a band at 1072 to 1052 cm^−1^ and at 1050 to 1016 cm^−1^, encompassing a region usually associated with vibrations of the O—O—H group.

No bands were noted which could be correlated with the absence (group 6) or presence (group 7) of the pyranoid ring.

The effects, on the spectra, of changing the anomeric group from hydroxyl to (a) acetamido and (b) methoxyl (with all other hydroxyl groups acetylated) are summarized in table 3. For comparison, the bands recorded for tetrahydropyran [4] are also listed.

Only one anomeric pair of *N*-glycopyranosylacetamides (compounds 10 and 11) was available for intercomparison; bands differentiating between these anomers are listed in table 4.

Barker and coworkers [5] mentioned that compound 17 (in a Nujol mull) showed bands at 929, 914, 898, 889, 878, and 759 cm^−1^; in this region, our spectrogram 17 (potassium chloride pellet) shows bands at 937, 910, 893, 878, and 768 cm^−1^. The spectra of compounds 18 to 21 in chloroform and in dioxane have been presented previously [6].

**Figure 1:**
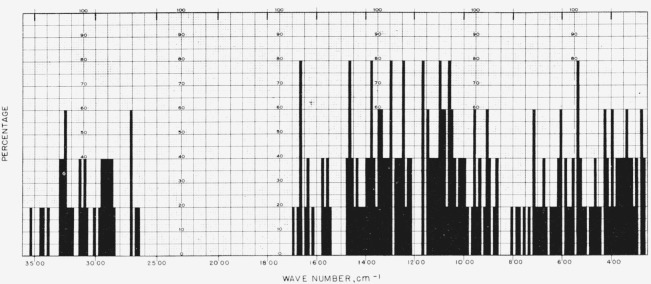
Percentage, of the *N*-acetylglycosylamines (group 1a), which showed absorption at the various regions of the infrared spectrum.

**Figure 2:**
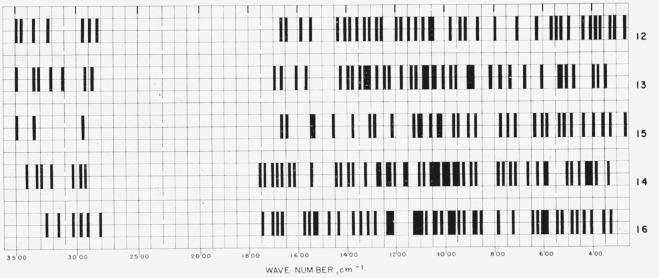
Positions of bands (cm^−1^) in the infrared absorption spectra of compounds 12 to 16 (groups 1c to 1f).

**Figure 3:**
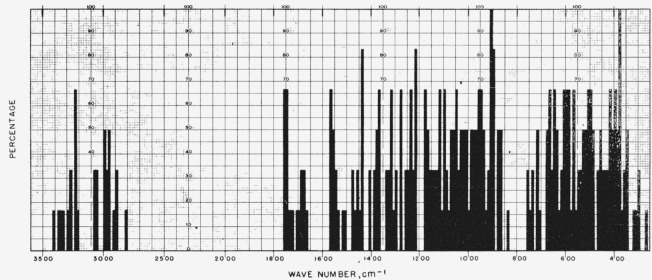
Percentage, of the acetate esters of *N*-acetylglycosylamines (group 1b), which showed absorption at the various regions of the infrared spectrum.

**Figure 4:**
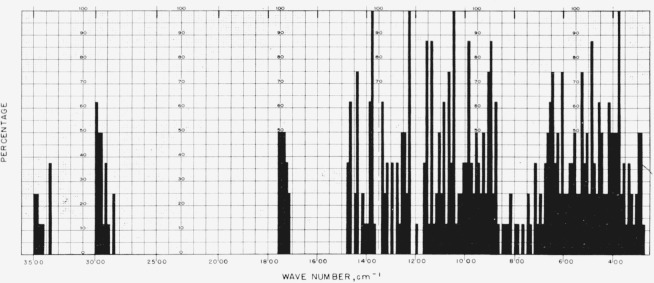
Percentage, of the reducing, pyranose acetates (group 5a), which showed absorption at the various regions of the infrared spectrum.

**Figure 5:**
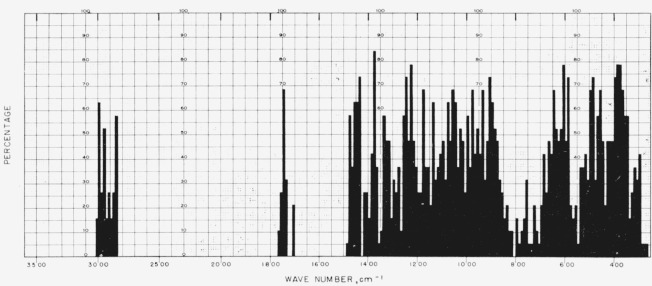
Percentage, of the acetylated methyl aldopyranosides of nineteen monosaccharides, which showed absorption at the various regions of the infrared spectrum (calculated from data in ref. [3]).

Figure 1 gives the percentage of the *N*-acetylglycosylamines (group 1a) that show absorption bands in the various regions of the infrared spectrum, and figure 3 provides the same kind of information for their acetate esters (group 1b); for convenient comparison, figure 2 shows the positions of bands (cm^−1^) in the infrared absorption spectra of the compounds in groups 1c to 1f. Figures 4 and 5 give the percentages of the reducing, pyranose acetates (group 5a) and of the acetylated methyl aldopyranosides of 19 monosaccharides [3], respectively, that show absorption bands in the various regions of the infrared spectrum. For the range of 5000 to 2000 cm^−1^ in figures 1 to 5, decrements of 20 cm^−1^ in wavenumber were used; and, for the range of 2000 to 250 cm^−1^, decrements of 10 cm^−1^.

## 6. Experimental Procedures

### 6.1. Preparation and Purification of the Compounds

The compounds listed in table 1 were prepared by the methods given in the references cited. The compounds were prepared in the course of earlier studies on the ring structure of *N*-glycosylacetamides, [7,8] and reducing, pyranose acetates [9]. Each compound was recrystallized from an appropriate solvent until further recrystallization caused no change in its melting point or optical rotation.

### 6.2. Preparation of the Pellets

Samples for spectrophotometric study were prepared in the solid phase, as pellets consisting of the crystalline compound suspended in an alkali-metal halide, exactly as previously described [2]. For the range of 5000 to 667 cm^−1^, a concentration of 0.4 mg of compound per 100 mg of potassium chloride was used; a concentration of 2 mg per 100 mg was also used for compound 16. For the range of 667 to 250 cm^−1^, a concentration of 2 mg of compound per 100 mg of potassium iodide was used, except for compound 4 (0.67 mg per 100 mg). Comparisons of intensity of absorption, from one compound to another can only be true and quantitative where the molar concentration is the same.

### 6.3. Measurement of Infrared Absorption

**Figure 6:**
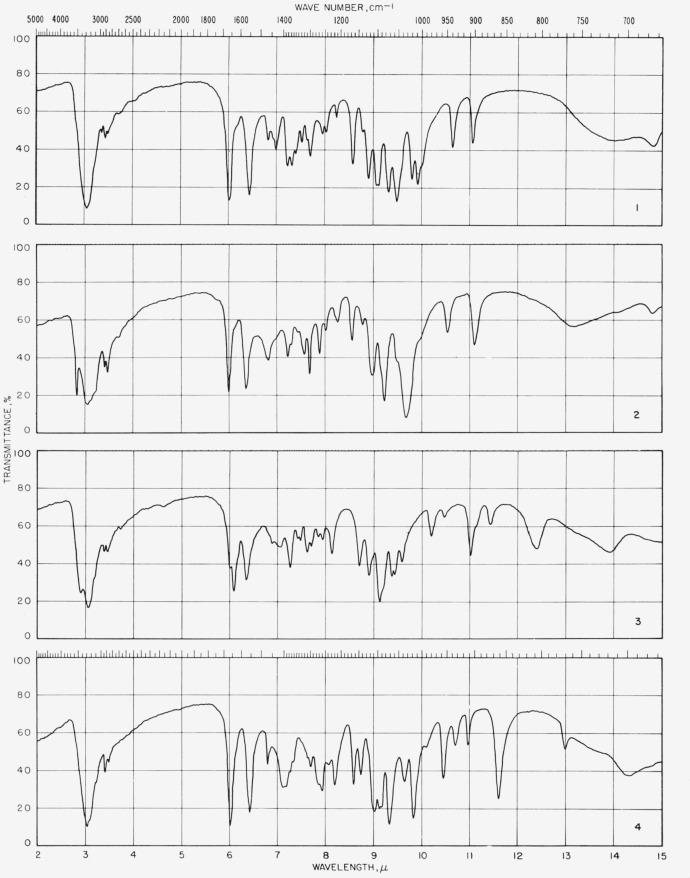

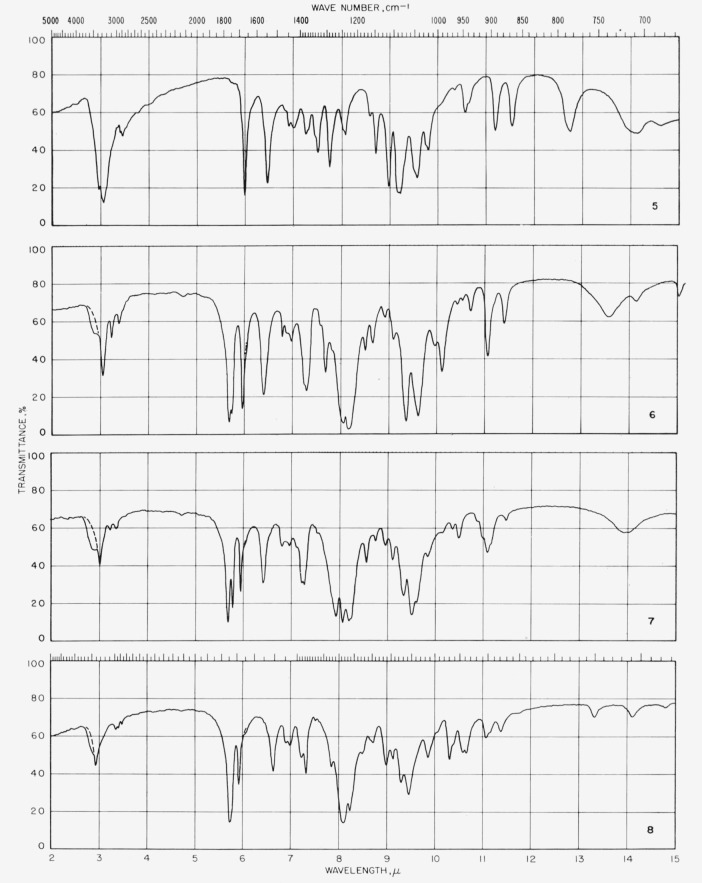

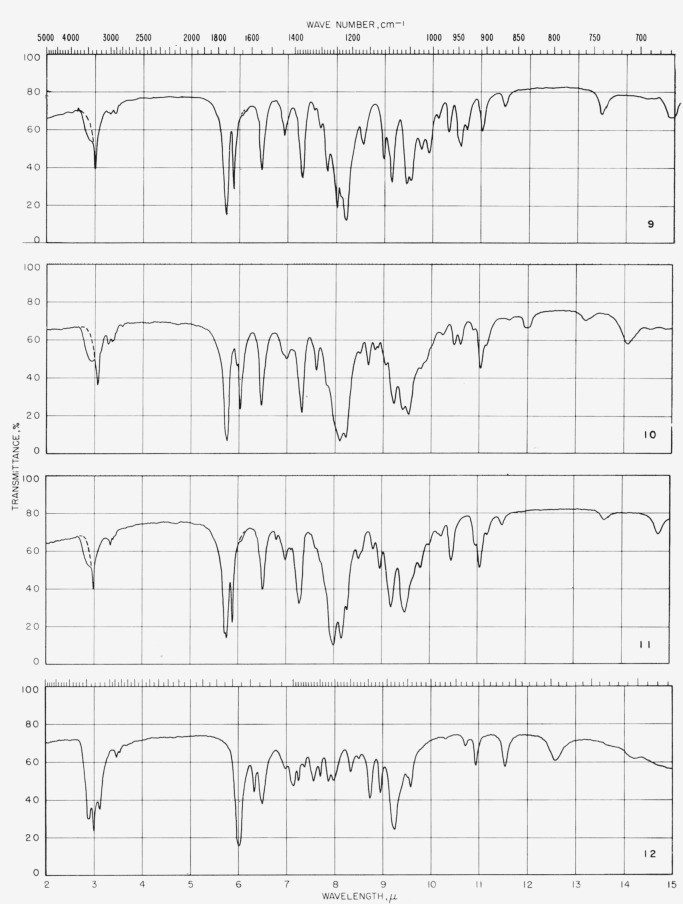

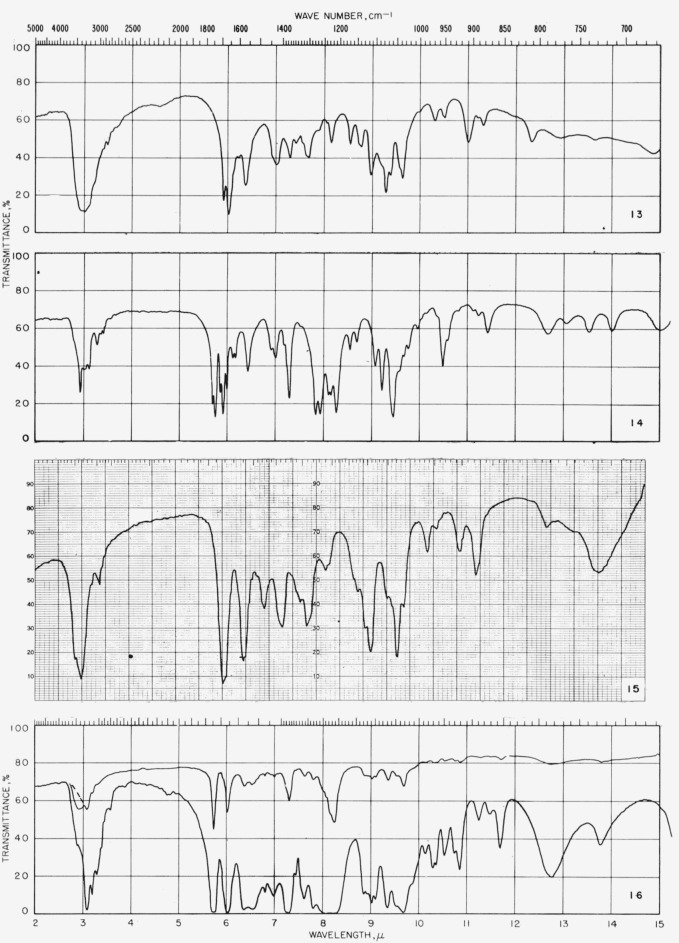

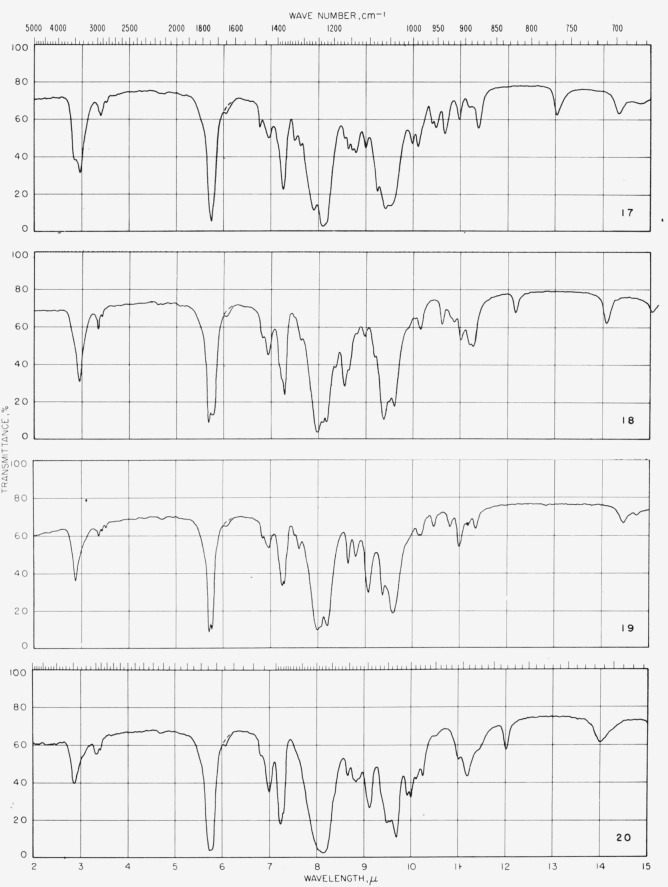

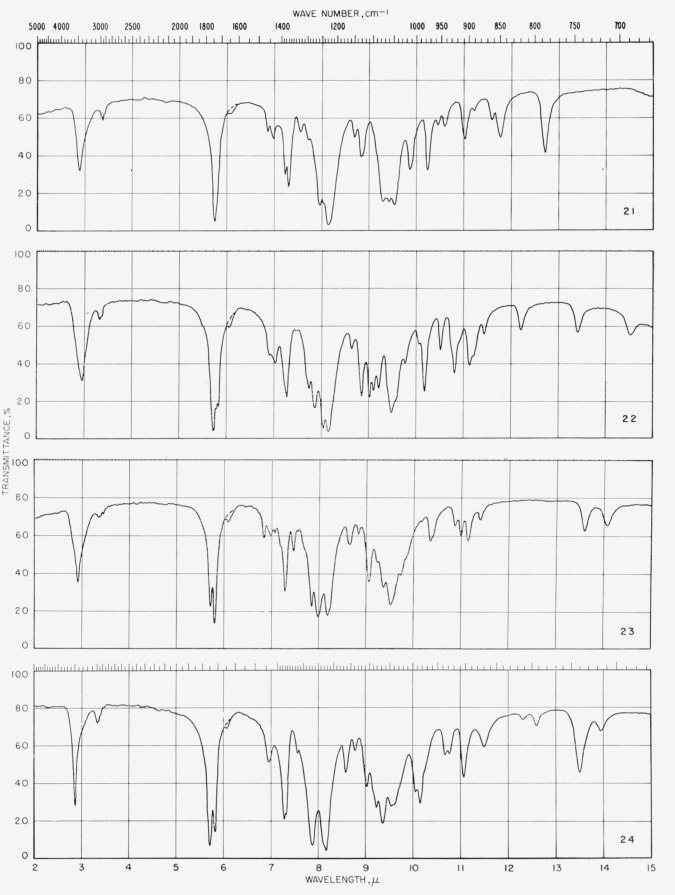
Spectrograms of materials in potassium chloride pellets. **1**, *N*-Acetyl-*β*-d-xylopyranosylamine; **2**, *N*-acetyl-*β*-d-glucopyranosylamine; **3**, *N*-acetyl-*β*-d-mannopyranosylamine, monohydrate; **4**, *N*-acetyl-*α*-l-arabinopyranosylamine. **5**, *N*-acetyl-*β*-d-galactopyranosylamine; **6**, *N*-acetyl-2,3,4-tri-*O*-acetyl-*β*-d-xy]osylamine; **7**, *N*-acetyl-2,3,4,6-tetra-*O*-acetyl-*β*-d-glueosylamine; **8**, *N*-acetyl-2,3,4,6-tetra-*O*-acetyl-*β*-d-mannosylamine. **9**, *N*-acetyl-2,3,4-tri-*O*-acetyl-*α*-l-arabinosylamine; **10**, *N*-acetyl-2,3,4,6-tetra-*O*-acetyl-*α*-d-galactosylamine; **11**, *N*-acetyl-2,3,4,6-tetra-*O*-acetyl-*β*-d-galactosylamine; **12**, -acetamido-1-deoxy-?-d-mannopyranuronamide. **13**, 1-acetamido-1-deoxy-?-d-galactopyranuronamide, pentahydrate; **14**, 1-acetamido-2,3,4-tri-*O*-acetyl-1-deoxy-?-d-mannuronamide; **15**, 1,1-bis(acetamido)-1-deoxy l-arabinitol; **16**, 1,1-bis(acetamido)-2,3,4,5-tetra-*O*-acetyl-1-deoxy-l-arabinitol. **17**, 2,3,4-tri-O-acetyl-*α*-d-xylopyranose; **18**, 1,2,3,4-tetra-*O*-acetyl-*α*-l-*xylo*-hexulopyranose; **19**, 2,3,4,6-tetra-*O*-acetyl-*β*-d-glucopyranose; **20**, 1,3,4,5,7-penta-*O*-acetyl-*α*-d-*gluco*-heptulopyranose. **21**, 2,3,4,6-tetra-*O*-acetyl-*α*-d-mannopyranose; **22**, 1,3,4,5-tetra-*O*-acetyl-*β*-d-*arabino*-hexulopyranose; **23**, 2,3,4,6-tetra-*O*-acetyl-*β*-d-galactopyranose; **24**, 2,3,4,6-tetra-*O*-acetyl-*α*-d-talopyranose.

**Figure 7:**
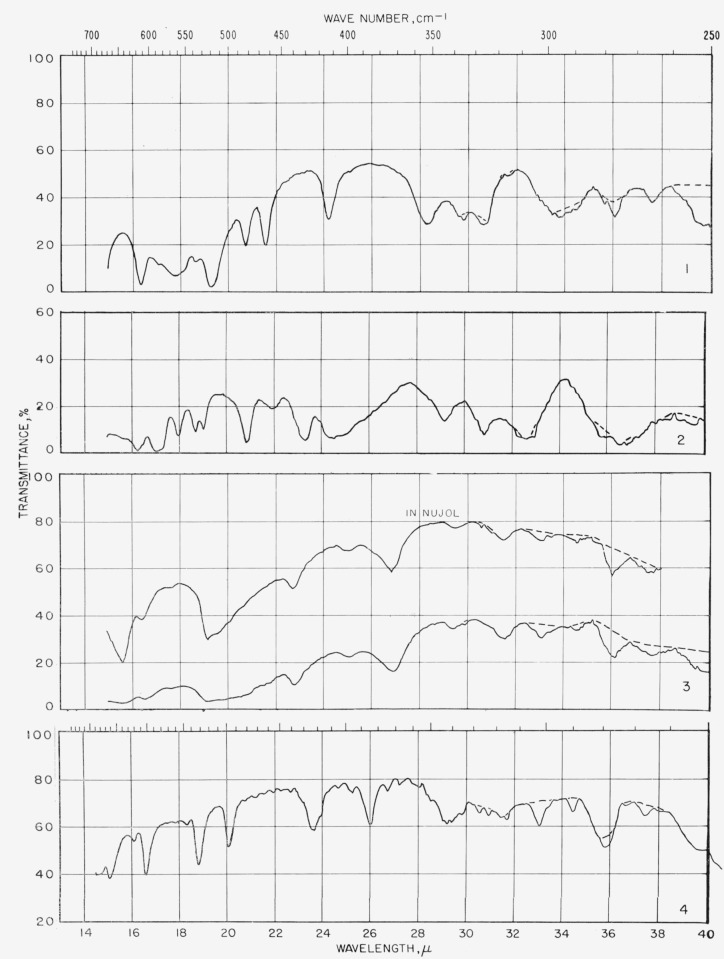

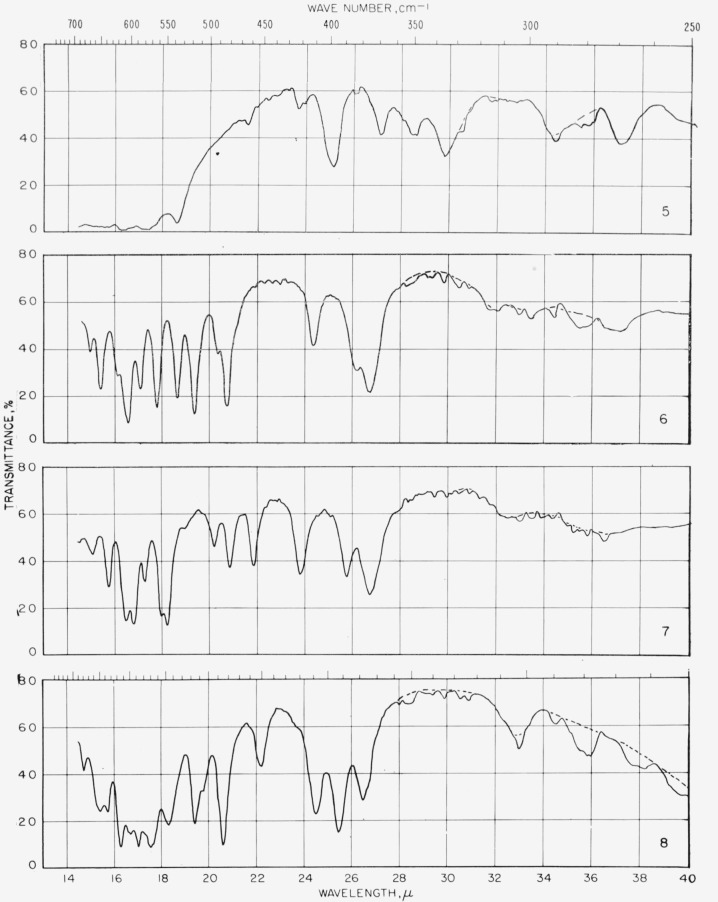

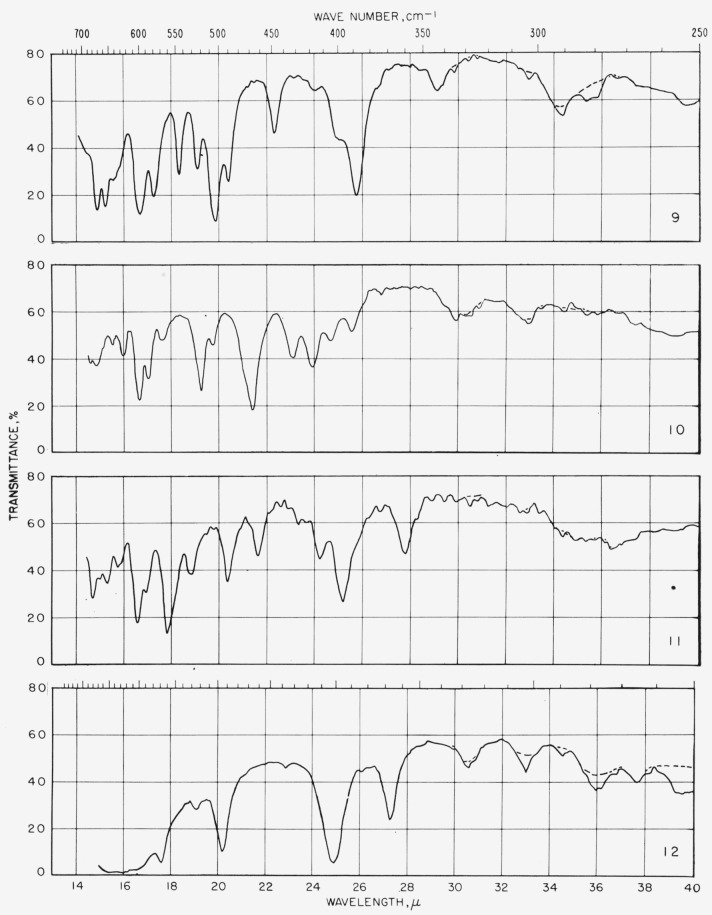

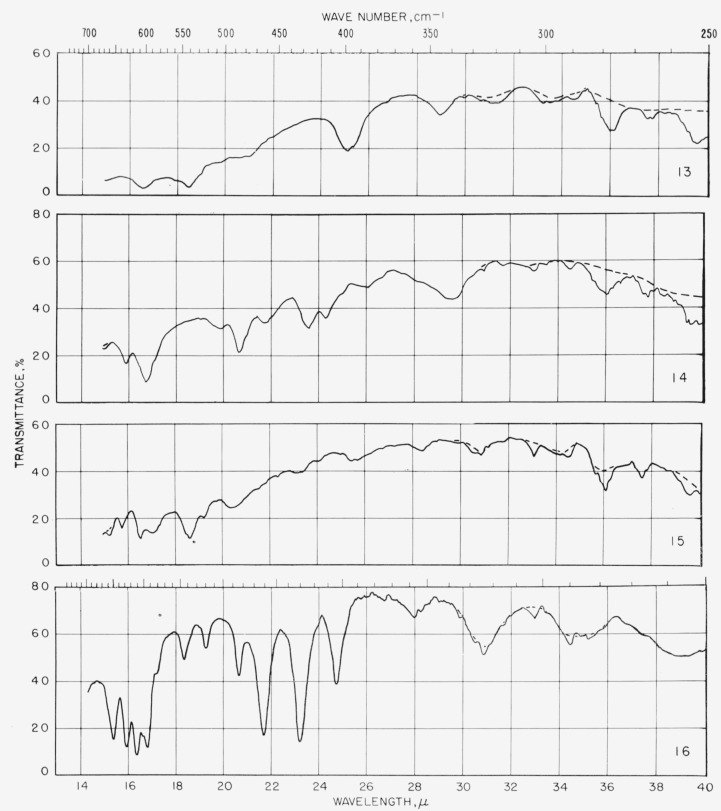

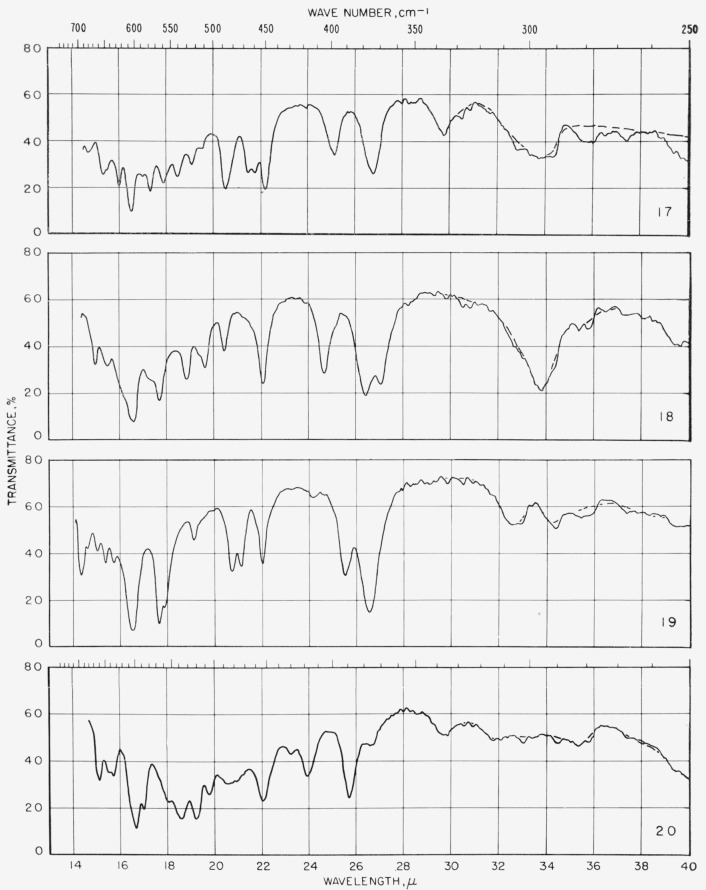

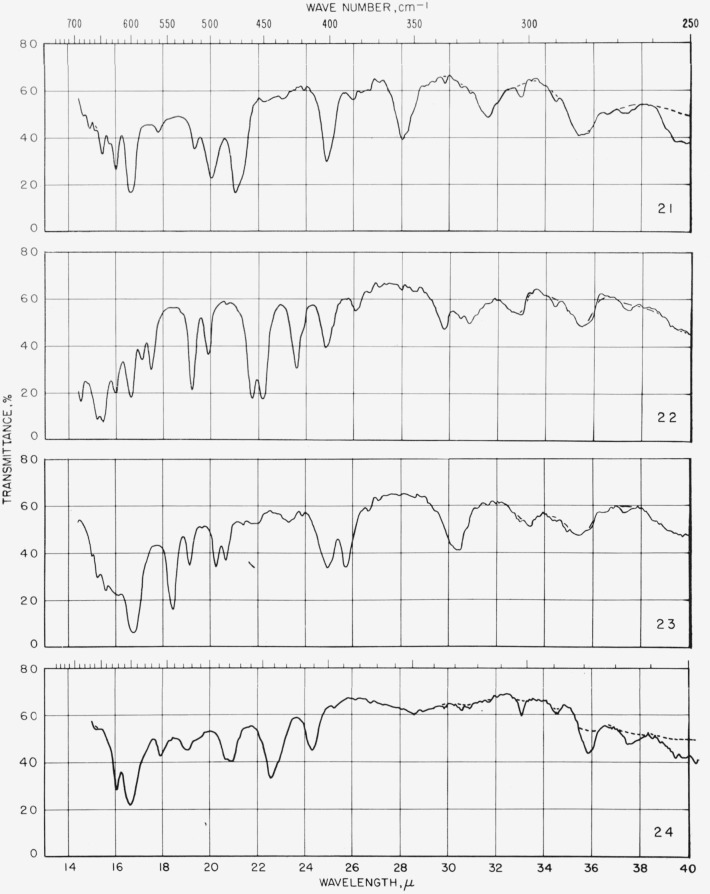
Spectrograms of materials in potassium iodide pellets. **1**, *N*-Acety-*β*-d-xylopyranosylainine; **2**, *N*-acetyl-*β*-d-glucopyranosylamine; **3**, *N*-acetyl-*β*-d-mannopyranosylamine, monohydrate; **4**, *N*-acetyl-a-l-arabinopyranosylamine. **5**, *N*-acetyl-*β*-d-galactopyranosylamine; **6**, *N*-acetyl-2,3,4,tri-*O*-acetyl-*β*-d-xylosylamine; **7**, *N*-acetyl-2,3,4,6-tetra-*O*-acetyl-*β*-d-glucosylamine; **8**, *N*-acetyl-2,3,4,6-tetra-*O*-acetyl-*β*-d-mannosylamine. **9**, *N*-acetyl-2,3,4-tri-*O*-acetyl-*α*-l-arabinosylamine; **10**, *N*-acetyl-2,3,4,6-tetra-*O*-acetyl-*α*-d-galactosylamine; **11**, *N*-acetyl-2,3,4,6-tetra-*O*-acetyl-*β*-d-galactosylamine; **12**, 1-acetamido-1-deoxy-?-d-mannopyranuronamide. **13**, 1-acetamido-1-deoxy-?-d-galactopyranuronamide, pentahydrate; **14**, 1-acetaraido-2,3,4-tri-*O*-acetyl-1-deoxy-?-d-mannuronamide; **15**, 1,1-bis(acetamido)-1-deoxy-l-arabinitol; **16**, 1,1-bis(acetamido)-2,3,4,5-tetra-*O*-acetyl-1-deoxy-l-arabinitol. **17**, 2,3,4-tri-*O*-acetyl-*α*-d-xylopyranose; **18**, 1,3,4,5-tetra-*O*-acetyl-*α*-l-*xylo*-hexulopyranose; **19**, 2,3,4,6-tetra-*O*-acetyl-*β*-d-glucopyranose; **20**, 1,3,4,5,7-penta-*O*-acetyl-*α*-d-*gluco*-heptulopyranose. **21**, 2,3,4,6-tetra-*O*-acetyl-*α*-d-mannopyranose; **22**, 1,3,4,5-tetra-*O*-acetyl-*β*-d-*arabino*-hexulopyranose; **23**, 2,3,4,6-tetra-*O*-acetyl-*β*-d-galactopyranose; **24**, 2,3,4,6-tetra-*O*-acetyl-*α*-d-talopyranose.

The spectrograms are shown in figures 6 and 7. That in figure 6 for compound 15 was recorded with a Beckman Model IR4 (double beam) spectrophotometer equipped with prisms of sodium chloride. The rest were recorded with a Perkin-Elmer Model 21 (double-beam) spectrophotometer equipped with a prism of sodium chloride (for the range of 5000 to 667 cm^−1^) and of cesium bromide (for the range of 667 to 250 cm^−1^), as previously described [2].

Some absorption attributable to water (in the compound, the alkali halide, or both) was observed at 3448 and 1639 cm^−1^ and, attributable to atmospheric water vapor, in the far-infrared curves. These regions are drawn on the spectrograms with dashed lines which are not to be interpreted quantitatively.

## Figures and Tables

**Table 1:** Compounds measured and index to spectrograms

Code	Compound	Reference	Spectrogram

A. *N*-GLYCOPYRANOSYLACETAMIDES			

	1. *Unsubstituted*		
10.11?82	*N*-Acetyl-*β*-d-xylopyranosylamine	1	1
10.21?82	*N*-Acetyl-*β*-d-glucopyranosylamine	1	2
10.22?8299	*N*-Acetyl-*β*-d-mannopyranosylamine, monohydrate.	1	3
10.13?82	*N*-Acetyl-*α*-l-arabinopyranosylamine	2	4
10.23?82	*N*-Acetyl-*β*-d-galactopyranosylamine	3	5
	2. *Acetate esters*		
12.11?82	*N*-Acetyl-2,3,4-tri-*O*-acetyl-*β*-d-xylosylamine	1	6
12.21?82	*N*-Acetyl-2,3,4,6-tetra-*O*-acetyl-*β*-d-glucosylamine.	1	7
12.22?82	*N*-Acetyl-2,3,4,6-tetra-*O*-acetyl-*β*-d-mannosyl amine.	1	8
12.13?82	*N*-Acetyl-2,3,4-tri-*O*-acetyl-*α*-l-arabinosylamine	2	9
12.23?82	*N*-Acetyl-2,3,4,6-tetra-*O*-acetyl-*α*-d-galactosylamine.	3	10
12.23?82	*N*-Acetyl-2,3,4,6-tetra-*O*-acetyl-*β*-d-galactosylamine.	3	11

B. *N*-(GLYCOPYRANOSYLURONAMIDE) ACETAMIDES			

10.22?8274	1-Aectamido-1-deoxy-?-d-mannopyranuronamide	4	12
10.23?827499	1-Acetamido-1-deoxy-?-d-galactopyranuronamide pentahydrate.	5	13
12.22?8274	1-Acetamido-2,3,4-tri-*O*-acetyl-1-deoxy-?-d-mannuronamide.	4	14

C. 1,1-BIS(ACETAMIDO)-1-DEOXYALDITOLS			

10.13782	1,1-Bis(acetamido)-1-deoxy-l-arabinitol	6	15
12.1375282	1,1-Bis(acetamido)-2,3,4,5-tetra-*O*-acetyl-1-deoxy-l-arabinitol.	(a)	16

D. REDUCING, PYRANOSE ACETATES			

12.11?0	2,3,4-Tri-*O*-acetyl-*α*-d-xylopyranose	7	17
12.71?0	1,3 4,5-Tetra-*O*-acetyl-*α*-l-*xylo*-hexulopyranose	8	18
12.21?0	2,3,4,6-Tetra-*O*-acetyl-*β*-d-glucopyranose	9	19
12.81?0	1,3,4,5,7-Penta-*O*-acetyl-*α*-d-*gluco*-heptulopyranose.	10	20
12.22?0	2,3,4,6-Tetra-*O*-acetyl-*α*-d-mannopyranose	11	21
12.73?0	1,3,4,5-Tetra-*O*-acetyl-*β*-d-*arabino*-hexulopyranose.	12	22
12.23?0	2,3,4,6-Tetra-*O*-acetyl-*β*-d-galactopyranose	13	23
12.24?0	2,3,4,6-Tetra-*O*-acetyl-*α*-d-talopyranose	14	24

a Prepared by acetylating compound 15; the enantiomorph was described by R. C. Hockett, V. Deulofeu, A. L. Sedoff, and J. R. Mendive, J. Am. Chem. Soc. **60**, 278 (1938).

1. H. S. Isbell and H. L. Frush, J. Org. Chem. **23**, 1309 (1958).

2. H. S. Isbell and H. L. Frush, J. Research NBS **46**, 132 (1951) RP2186.

3. H. L. Frush and H. S. Isbell, J. Research NBS **47**, 239 (1951) RP2248.

4. H. L. Frush and H. S. Isbell, J. Research NBS **41**, 11 (1948) RP1898.

5. H. L. Frush and H. S. Isbell, J. Research NBS **41**, 609 (1948) RP1943.

6. H. S. Isbell and H. L. Frush, J. Am. Chem. Soc. **71** 1579 (1949).

7. C. S. Hudson and J. K. Dale, J. Am. Chem. Soc. **40**, 997 (1918).

8. H. H. Schlubach and G. Graefe, Liebigs Ann. Chem. **532**, 211 (1937).

9. S. B. Hendricks, O. R. Wulf, and U. Liddel, J. Am. Chem. Soc. **58**, 1997 (1936).

10. H. L. Frush and H. S. Isbell, J. Research NBS **35**, 111 (1945) RP1663.

11. P. A. Levene and R. S. Tipson, J. Biol. Chem. **90**, 89 (1931).

12. E. Pacsu and F. V. Rich, J. Am. Chem. Soc. **55**, 3018 (1933).

13. J. Compton and M. L. Wolfrom, J. Am. Chem. Soc. **56**, 1160 (1934).

14. W. W. Pigman and H. S. Isbell, J. Research NBS **19**, 189 (1937) RP1021.

**Table 2:** Structural groups studied

Group	Structural feature	Compounds (serial numbers) in group
		
1	Acetamido (amide, secondary)	1 to 16
1a	*N*-Acetyl	1 to 5
1b	*N*-Acetyl-*O*-acetyl	6 to 11
1c	*N*-Acetyl; amide (primary)	12, 13
1d	*N*-Acetyl-*O*-acetyl; amide (primary)	14
1e	*N*-Acetyl; open chain	15
1f	*N*-Acetyl-*O*-acetyl; open chain	16
2	*O*-Acetyl	6 to 11, 14, 16, 17 to 24
3	Amide, primary	12 to 14
4	Hydrate	3, 13
5	Hydroxyl group	1 to 5, 12, 13, 15, 17 to 24
5a	Anomeric hydroxyl group, only	17 to 24
6	Open chain	15, 16
7	Pyranoid ring	1 to 14, 17 to 24

**Table 3:** Effect of changing the anomeric group from hydroxyl to (a) acetamido and (b) methoxyl; and comparison with hands of tetrahydropyran

Bands (cm^−1^) of tetrahydropyran^a^	Spectral range of bands (cm^−1^) shown by every member of the group of compounds		
	Hydroxyl (group 5a, table 2)	Acetamido (group 1b, table 2)	Methoxyl (see ref. [3])
			
……………………………	3597 to 3367	……………………………	……………………………
……………………………	……………………………	3378 to 3268	……………………………
……………………………	……………………………	or 3356 to 3236	……………………………
……………………………	2994 to 2976	2994 to 2950	2994 to 2950
……………………………	2959 to 2907	or 2976 to 2907	$\{\begin{array}{c} \text{or}\;2985\;\text{to}\;2941\;\\ \text{or}\;2976\;\text{to}\;2907 \end{array}$
……………………………	……………………………	……………………………	or 2915 to 2841
……………………………	……………………………	……………………………	……………………………
……………………………	1757 to 1742	$\}\begin{array}{c} \;1751\;\text{to}\;1742\;\\ \text{or}\;1742\;\text{to}\;1727 \end{array}$	1751 to 1736
……………………………	or 1751 to 1736		……………………………
……………………………	or 1742 to 1718		……………………………
……………………………	……………………………	1701 to 1675	……………………………
……………………………	……………………………	1565 to 1555	……………………………
……………………………	……………………………	or 1560 to 1536	……………………………
……………………………	1477 to 1462	……………………………	1473 to 1456
……………………………	……………………………	……………………………	or 1462 to 1447
1451	1449 to 1433	1441 to 1433	1443 to 1433
1381	1385 to 1374	……………………………	1385 to 1372
……………………………	or 1377 to 1372	1374 to 1368	or 1379 to 1368
1348	b1340 to 1323	$\{\;\begin{array}{c} 1333\;\text{to}\;1316\\ \text{or}\;1325\;\text{to}\;1312 \end{array}\;$	1348 to 1321
			or 1335 to 1318
1296	……………………………	1299 to 1258	……………………………
1272	1271 to 1247	or 1279 to 1253	1264 to 1247
1256	……………………………	or 1253 to 1238	or 1256 to 1239
……………………………	……………………………	or 1241 to 1229	……………………………
……………………………	1230 to 1221	or 1238 to 1224	1236 to 1221
……………………………	……………………………	or 1229 to 1217	or 1227 to 1217
1202	……………………………	or 1224 to 1211	or 1224 to 1202
……………………………	……………………………	……………………………	1200 to 1172
……………………………	……………………………	1179 to 1166	or 1190 to 1167
1160	1166 to 1152	or 1168 to 1149	……………………………
……………………………	1140 to 1127	or 1126 to 1114	1148 to 1115
……………………………	……………………………	(or 1122 to 1106)	……………………………
……………………………	……………………………	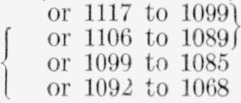	1114 to 1086
1097	1105 to 1076		} or 1107 to 1074
……………………………	1070 to 1054	$\{\;\begin{array}{c} \text{or}\;1070\;\text{to}\;1056\\ \text{or}\;1068\;\text{to}\;1049 \end{array}$	1068 to 1052 or 1060 to 1042
1050	1050 to 1041	or 1059 to 1041	} or 1026 to 1022
1033	……………………………	or 1041 to 1016	
……………………………	……………………………	or 1021 to 1005	or 1026 to 1006
1012	……………………………	(or 1016 to 1003)	……………………………
……………………………	……………………………	or 1014 to 996	} 1006 to 977
……………………………	……………………………	or 996 to 979	
……………………………	989 to 978	or 989 to 972	} or 984 to 951
969	……………………………	967 to 955	
……………………………	……………………………	or 965 to 948	……………………………
……………………………	962 to 938	or 958 to 947	} or 958 to 933
……………………………	or 952 to 921	940 to 914	
……………………………	911 to 898	909 to 904	917 to 903
……………………………	(or 910 to 896)	(or 907 to 902)	……………………………
……………………………	or 907 to 893	……………………………	or 907 to 897
……………………………	(or 905 to 891)	……………………………	……………………………
……………………………	……………………………	……………………………	898 to 875
875,856,818	887 to 864	880 to 862	or 889 to 867
……………………………	……………………………	741 to 711	……………………………
……………………………	……………………………	678 to 665	……………………………
……………………………	676 to 656	or 673 to 648	666 to 648
……………………………	……………………………	(or 665 to 645)	……………………………
……………………………	656 to 642	or 655 to 637	or 660 to 638
……………………………	……………………………	or 649 to 635	or 649 to 626
……………………………	636 to 615	……………………………	or 638 to 619
……………………………	……………………………	615 to 600	or 622 to 603
……………………………	……………………………	(or 608 to 599)	……………………………
……………………………	604 to 597	or 605 to 597	or 612 to 597
……………………………	……………………………	or 600 to 587	or 602 to 577
……………………………	……………………………	or 591 to 579	or 596 to 571
……………………………	……………………………	or 581 to 561	……………………………
……………………………	……………………………	(or 579 to 558)	……………………………
……………………………	571 to 544	or 570 to 546	……………………………
……………………………	or 562 to 522	546 to 522	……………………………
……………………………	525 to 516	$\{\;\begin{array}{c} \text{or}\;533\;\text{to}\;516\\ \left(\text{or}\;529\;\text{to}\;515\right) \end{array}\;$	……………………………
……………………………	……………………………	509 to 493	} 520 to 491
……………………………	……………………………	……………………………	or 499 to 482
……………………………	489 to 476	……………………………	or 491 to 475
……………………………	……………………………	……………………………	470 to 451
……………………………	455 to 442	……………………………	……………………………
……………………………	……………………………	420 to 409	} 428 to 399
……………………………	419 to 402	$\{\;\begin{array}{c} \left(\text{or}\;417\;\text{to}\;408\right)\\ \text{or}\;414\;\text{to}\;400 \end{array}$	
……………………………	or 403 to 390	(or 412 to 398)	……………………………
……………………………	or 398 to 384	or 398 to 383	……………………………
……………………………	379 to 374	379 to 375	376 to 367
……………………………	296 to 279	……………………………	……………………………

a Bands recorded by Burket and Badger [4].

b Except compound 20.

**Table 4:** Bands (cm^−1^) differentiating between the anomers of *N*-acetyl-2,8,4,6-tetra-*O*-acetyl-*d*-galactosylamine

10 (*α*)	11 (*β*)	10 (*α*)	11 (*β*)
			
3215	………	………	1408
3058	………	………	1376
2801	………	………	1333
1664	………	………	1253
1106	………	………	1037
1064	………	………	870
943	………	………	667
834	………	………	533
711	………	………	493
307	………	………	452
………	2941	………	445
………	2890	………	430
………	1751	………	361
………	1513	………	355
………	1473	………	348
………		………	341

## Footnotes

1 Figures in brackets indicate the literature references at the end of this paper. The references for table 1 are given at the end of the table.
